# Naturally acquired antibodies to *Plasmodium vivax* Pv_LISP-2, a potential liver stage vaccine antigen

**DOI:** 10.3389/fimmu.2026.1763514

**Published:** 2026-03-27

**Authors:** Rafael Amaral Donassolo, Gisele Tatiane Soares da Veiga, Líndice Mitie Nisimura, Najara Carneiro Bittencourt, Yanka Evellyn Alves R. Salazar, Dayanne Kamylla Alves da Silva Barros, Stefanie Costa Pinto Lopes, Tais Nobrega de Sousa, Fabio Trindade Maranhão Costa, Letusa Albrecht

**Affiliations:** 1 Laboratório de Pesquisa em Apicomplexa – Instituto Carlos Chagas, Fundação Oswaldo Cruz, Curitiba, Paraná, Brazil; 2 Grupo de Pesquisa em Imunologia Molecular, Celular e Inteligência Artificial, Instituto Carlos Chagas, Fundação Oswaldo Cruz, Curitiba, Paraná, Brazil; 3 Laboratório de Doenças Tropicais Prof. Dr. Luiz Jacintho da Silva, Departamento de Genética, Evolução, Microbiologia e Imunologia, Universidade de Campinas - UNICAMP, Campinas, Brazil; 4 Biologia Molecular e Imunologia da Malária, Instituto René Rachou, Fundação Oswaldo Cruz (Fiocruz), Belo Horizonte, MG, Brazil; 5 Instituto Leônidas & Maria Deane, Fundação Oswaldo Cruz (Fiocruz), Manaus, AM, Brazil; 6 Fundação de Medicina Tropical Doutor Heitor Vieira Dourado (FMT-HVD), Manaus, AM, Brazil; 7 Department of Microbiology, Tumor and Cell Biology, Karolinska Institutet, Solna, Sweden

**Keywords:** antigenicity, liver stage, malaria, plasmodium vivax, vaccine candidate

## Abstract

**Introduction:**

*Plasmodium vivax* is responsible for most malaria cases in Latin America and Southeast Asia and can cause severe manifestations. Prevention strategies such as vaccines, have been hampered by the complexity of the parasite’s life cycle, especially in the liver stages. The *P. vivax*-Liver-Specific Protein 2 (Pv_LISP-2) has been described as essential for intrahepatic development, but its antigenicity has not yet been explored. Thus, in this study we evaluate the naturally acquired humoral response against two regions of Pv_LISP-2 in populations from the Brazilian Amazon exposed to *P. vivax* infection. Plasma samples were collected from infected individuals in Manaus (Amazonas state) and Boa Vista (Roraima state).

**Methods:**

Two structural regions of Pv_LISP-2 (Pv_L.seq1 and Pv_L.seq2) were expressed in *E. coli*, purified, and applied in ELISA assays to detect total IgM, IgG, and IgG subclasses antibodies. Furthermore, longitudinal follow-up was conducted for up to 180 days in part of the Manaus cohort. Pv_LISP-2 fragments were recognized by antibodies of *P. vivax-*infected individuals. During the acute phase, the response was characterized by high levels of IgM, followed by the induction of IgG, especially against Pv_L.seq1. The cytophilic subclasses IgG1 and IgG3 were the predominant subclasses. IgM responses correlated with parasitemia and days of symptoms but showed a negative association with hematological parameters (hemoglobin, hematocrit, and erythrocytes).

**Discussion:**

This is the first study to evaluate naturally acquired antibodies against this liver-stage antigen of *P. vivax*, we showed that Pv_LISP-2 is naturally antigenic in individuals exposed to vivax malaria. While the functional implications of these responses remain to be fully elucidated, our findings support further investigation of Pv_LISP-2 as a potential vaccine candidate targeting the liver stage of *P. vivax*.

## 1 Introduction

Malaria remains one of the deadliest infectious diseases worldwide. In 2023, it was reported 247 million malaria cases and around 619.000 deaths. In Brazil, *P. vivax* is responsible for more than 85% of the malaria cases and has been associated with severe manifestations, including renal failure, acute respiratory distress syndrome, severe anemia, and other clinical complications (1–3). Furthermore, due to its complex life cycle, *P. vivax* is considered as the most difficult species to eliminate, and preventive strategies need to be developed aiming at malaria eradication (4).

The life cycle of the parasite consists of a pre-erythrocytic, erythrocytic, and sexual stage. The pre-erythrocytic stage begins when a female *Anopheles* mosquito inoculates sporozoites into the bloodstream; these rapidly migrate to the liver, invade hepatocytes, and undergo schizogony, giving rise to thousands of merozoites (5). Merozoites are subsequently released into the bloodstream and invade red blood cells, preferentially reticulocytes, initiating the erythrocytic stage. During intraerythrocytic development, parasites progress through ring, trophozoite, and schizont stages, culminating in synchronized red blood cell rupture and release of new merozoites, which drives parasite amplification and the clinical manifestations of malaria (6, 7). In addition, *P. vivax* has the ability to form dormant hypnozoites which can cause several relapses of the disease, and it is believed that relapses contribute up to 80% of all blood-stage infections (8, 9). The greatest progress towards malaria eradication will require complete elimination of these forms or reducing the number of relapses that are the main driver of the disease.

Thus, attacking the pre-erythrocyte and liver stages is crucial to impair pathogen development, may be critical to reduce incidence or reinfections of the disease and stop its clinical manifestations (10). In this scenario, antibody-mediated protection against malaria acts mainly by limiting parasite burden rather than completely preventing infection. Antibodies can neutralize sporozoites and block hepatocyte invasion, inhibit merozoite entry into red blood cells, and promote Fc-dependent mechanisms such as opsonic phagocytosis and complement activation (11–14). In this context, vaccine development represents one of the most important tools for malaria prevention and control. The vaccine candidates against *Plasmodium* spp. are basically composed by blood-stage proteins, and the most studied is the Circumsporozoite Protein (CSP). This antigen has been tested in a vaccine formulation against *P. falciparum* in children from Africa and showed promising results (15, 16). However, for *P. vivax*, just a few vaccine candidates have reached clinical trials, and most have shown low efficacy.

One of the most important approaches regarding the development of prevention tools is to understand the protective immunity and characterize immune responses in a natural infection. The knowledge of the antibody profile of individuals exposed to a specific antigen in a malaria-endemic area can provide useful information concerning malaria vaccine development. However, antigens specific to liver stage are poorly studied and just a few information about anti-Plasmodial immunity of liver stages is available.

The Liver Specific Antigen-2 (Pv_LISP-2) is a 6-cys family protein and was discovered as a membrane adhesin, involved in the adhesion of infected red blood cells to host endothelium and later it was found to be crucial to merozoite development (17, 18). Pv_LISP-2 is abundantly expressed in the liver stage of different species of *Plasmodium* spp. and starts quickly after infection, increasing during schizogony (19). So far, there is a lack of studies regarding the naturally acquired humoral response against Pv_LISP-2 in individuals exposed to *P. vivax* infection. Here, we evaluated seroprevalence to the Pv_LISP-2 antigen in *P. vivax*-infected subjects in two different malaria-endemic areas of Brazil, and then we correlated hematological and immunological parameters associated with the antibody response. Our results can contribute to the development of future strategies against liver stage blocking strategies.

## 2 Methodology

### 2.1 Protein selection, cloning, expression and purification

**Figure 1:**
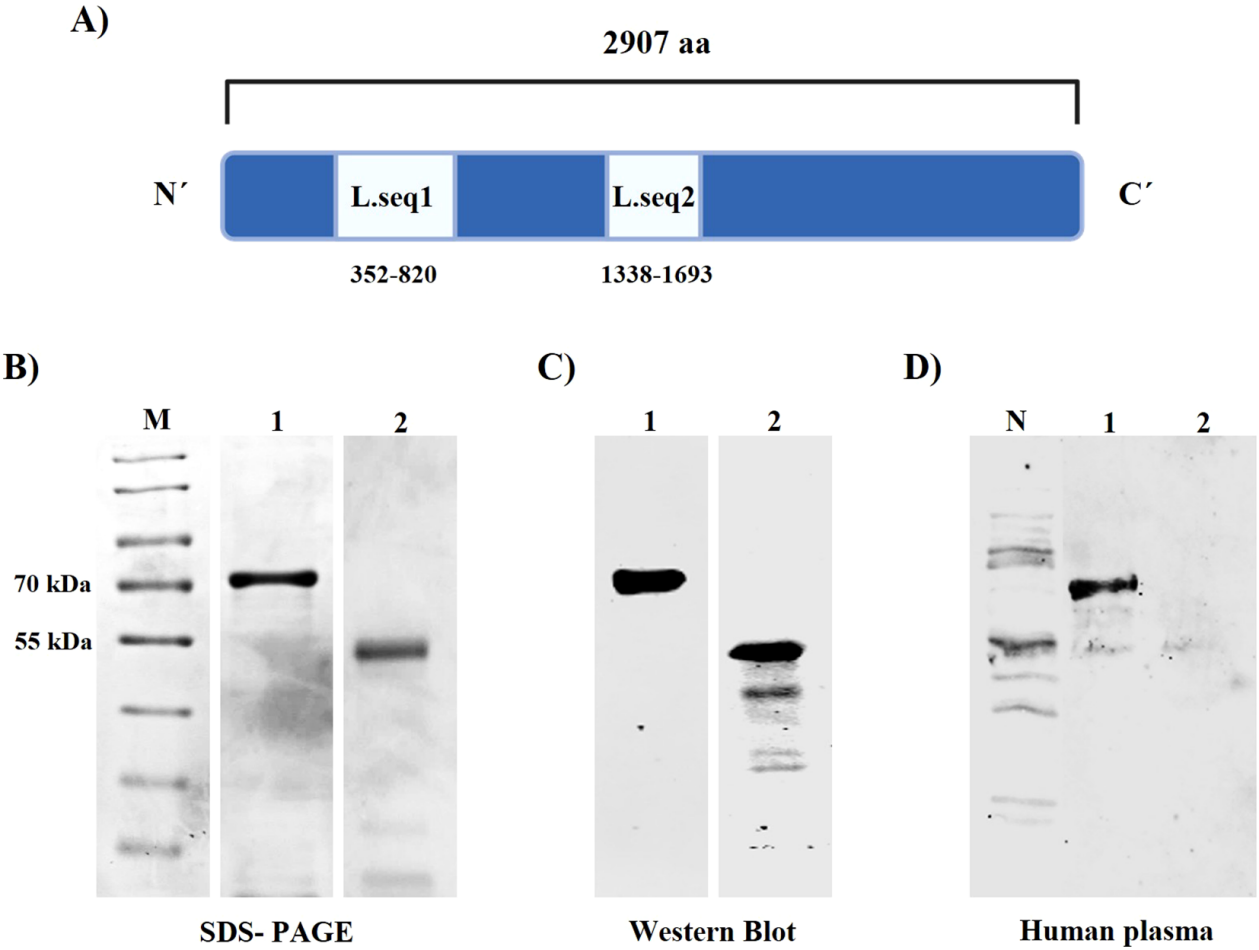
Schematic representation of the LISP-2 protein structure and analysis of the production and purification of recombinant proteins in *E. coli* SHuffle. The two fragments indicated in the structure **(A)** were expressed and after expression the culture was centrifuged, fractionated, lysed, and purified by chromatography using Akta purifier. The figure shows in **(B)** SDS-PAGE and in **(C)** western blot using anti-HIS6x. **(D)** Western blot using pool of plasma samples from P.vivax LISP-2 positive individuals evaluated by ELISA assay. M: Protein Marker; N- *E. coli* SHuflle T7 (negative control); 1-L.seq1; 2- L.seq2.

The gene encoding Liver-Specific Protein 2 (PvLISP-2) was retrieved from PlasmoDB under accession number PVX_000975. Two regions of the protein were selected for analysis: the N-terminal region (amino acids 352–820; designated PvLISP2_Seq1) and a repeat-containing region (amino acids 1338–1693; designated PvLISP2_Seq2) (18, 20) (**Figure 1A**). Both sequences were subjected to *in silico* antigenicity analysis using the VaxiJen tool (https://www.ddg-pharmfac.net/vaxijen/VaxiJen/VaxiJen.html) (considering score >0.5). The selected genes were synthesized by Thermo Scientific™ and cloned into the prokaryotic expression vector pRSET-A, containing an N-terminal 6×His fusion tag. Proteins were expressed in *E. coli* SHuffle T7 which was then cultured in 1 L of LB containing ampicillin (100 μg/mL) at 30°C. When the culture reached an optical density (OD)_600_ of 0.8 cells was induced by the addition of 1 mM IPTG for 16 h at 25°C (21).

The culture was collected by centrifugation (6000 g, 10 min) and resuspended in 25 mL of Buffer A (20 mM NaH_2_PO_4_, 500 mM NaCl, 10 mM imidazole, pH 8) and 2 tablets of protease inhibitor cocktail Roche cOmplete™ Mini EDTA-free (Roche 04693159001) and then disrupted using an M-110L Pneumatic High Shear Fluid Processor (Microfluidcs). Cells were pelleted by centrifugation at 10000 g at 4 °C for 30 min and the soluble fraction was purified using nickel affinity chromatography on a HisTrap HP 1 mL column (Cytiva, 29051021) in the Akta Purifier™ equipment. Protein elution was performed by imidazole gradient elution using the buffer (20 mM NaH_2_PO_4_, 500 mM NaCl, 500 mM imidazole, pH 8). The second purification was performed using ion exchange chromatography. Affinity-purified samples were diluted to a final concentration of 50 mM NaCl in Tris-HCl buffer (50 mM) and subjected to a HiTrap SP 5 mL cation exchange column (Cytiva, 17115101). Elution was performed by increasing the salt concentration using the ion exchange buffer (50 mM Tris-HCl, 1 M NaCl, pH 8). Protein expression and purification were performed by SDS-PAGE and Western blotting using 6x-His Tag Monoclonal Antibody (HIS.H8) (Invitrogen, MA1-21315).

### 2.2 Ethical approvals, study sites, and sample collections

This study was conducted using samples from individuals of two different endemic populations of the Brazilian Amazon: Manaus (Amazonas) and Boa Vista (Roraima). Both the projects were approved by the ethical committee Manaus (Ethics Committee: 84250218.4.0000.0005) and Boa Vista (Ethics Committee: 2751310 and 2243058). This study’s ethical and methodological aspects were approved by the Ethics Committee of Research Involving Human Subjects of Institute René Rachou/Fiocruz (report no. 2.803.756 and no. 2.243.058). All participants signed a written informed consent form, and the next of kin, caretakers, or guardians signed on behalf of minors/children enrolled in the study.

Samples from Manaus were obtained from 40 symptomatic individuals who were diagnosed with malaria by blood smear at the Tropical Medicine Foundation Hospital. Blood samples of the individuals were collected on day 0 (D0), 50 (D50) and 180 (D180) days after confirmation of infection. *P. vivax* infection was confirmed through blood smear microscopy test and quantitative PCR (qPCR). After a positive diagnosis, all individuals were treated according to the Brazilian guidelines, and a complete blood count was performed using a Sysmex KX21N analyzer (Sysmex Corporation-Roche, Japan).

The Boa Vista population consisted of symptomatic patients (n= 98) with acute *P. vivax* who had experienced fever within the last 48 hours. The detection and quantification of parasite densities were determined by qPCR targeting the 18S rRNA and Pvs25 genes, expressed as copies/µL. For the negative control, plasma samples were collected from healthy individuals from non-endemic regions.

### 2.3 Humoral immune responses and IgG isotyping

Antibody binding to Pv_LISP-2 was evaluated in a set of plasma samples using a previously described enzyme-linked immunosorbent assay (ELISA) protocol, with minor modifications (22). Briefly, 96-well high-binding microplates (Microlong, Greiner Bio-One, Ref 655061) were coated with 250 ng/well (50 µL) of recombinant proteins diluted in carbonate-bicarbonate buffer (0.2 M carbonate-bicarbonate, pH 9.7) and incubated overnight at 4 °C. The next day, the plates were washed three times using PBST, followed by blocking solution (5% fat-free milk powder in PBST) and incubated for 1 h. After three washes, sera were diluted 1:100 (100 µL) in PBST, added to each well and incubated again for 1 h. Then, the plates were washed three times and a dilution of 1:4000 of IgG (ThermoScientific A18805) and 1:2000 of anti-human IgM conjugated with horseradish peroxidase (HRP) (Sigma A6907-1ml) (100 µL) was added to each well as secondary antibody diluted in PBST. For isotype antibodies mouse anti-human IgG1 HRP (clone HP6001, Southern Biotechnology), mouse anti-human IgG2 HRP (clone HP6002, Southern Biotechnology), mouse anti-human IgG3 HRP (clone HP6050, Southern Biotechnology) and mouse anti-human IgG4 HRP (clone HP6023, Southern Biotechnology), were used at dilution 1:1000. After a final three wash step, 100 µL of 3,3’,5,5’–tetramethylbenzidine peroxidase substrate solution (TMB SureBlue KPL™) was added and incubated for 10 min. The reaction was stopped with 1 M hydrochloric acid and the absorbance at 450 nm was measured with a microplate reader (Synergy H1 Hybrid Reader – Biotek). The values were normalized, and the cut-off was calculated as the mean plus three standard deviations of the negative plasma samples (from individuals who had never had malaria). The reactivity indices (RIs) were obtained by the ratio between the absorbance values of each sample and the cut-off value. The recognition of the recombinant protein was considered positive when the reactivity index was higher than 1.0.

### 2.4 Western blotting

Western blotting was performed as previously described (23), with minor modifications. A fraction of purified protein was subjected to electrophoresis in a 10% SDS-PAGE polyacrylamide gel. After electrophoresis, the proteins were transferred to a nitrocellulose membrane (Hybond C, Amersham Biosciences) at 20 V for 1 h. The membrane was then stained with Ponceau S solution (Sigma P-3504 - 0.5%; 1% glacial acetic acid), subsequently destained by washing with PBST and incubated in blocking solution (PBS; 0.05% Tween 20; 5% fat-free milk powder) for 1 h with gentle agitation at room temperature. After washing with PBST, the membrane was incubated with a pool of Pv_Lisp-2-positive human sample (dilution 1:100 in PBST) overnight at 4 °C. After 3 washes using PBST, the secondary anti-human IgG 633 antibody (dilution 1:1.000 with PBST) was added and incubated for 1 h, protected from light. Images were obtained using the Odyssey^®^ (LI-COR Biotech).

### 2.5 Statistical analysis

Statistical analyses were performed using the GraphPad Prism 8.4.3 software. Normality tests were done using the one-sample Kolmogorov–Smirnoff or Shapiro test. Correlations were tested using Spearman’s test for nonparametric data or Pearson for parametric data. Differences in antibody frequencies between two groups were tested by Fisher’s exact test. The magnitude of the reactivity indices was compared using the Kruskal–Wallis test followed by Dunn’s multiple-comparison *post hoc* test, or the Wilcoxon test, as appropriate. Longitudinal antibody responses were analyzed using linear mixed-effects models to account for repeated measurements within individuals. Time (days 0, 50, and 180), antibody isotype (IgG or IgM), and antigen were included as fixed effects. *Post hoc* multiple comparisons were performed using Benjamini–Hochberg test. *p* values < 0.05 were considered statistically significant.

## 3 Results

### 3.1 Clinical and epidemiological profile of the population studied

In the sample population from Manaus, at the moment of vivax malaria diagnosis (D0), all individuals presented at least one clinical symptom, most commonly fever, chills, and headache. The mean parasitemia was 2685 parasites/μL of blood (range: 1.910–3.461; 95% CI). Hematological analysis revealed that 40% (14/40) were anemic during the acute phase. In addition, 87.7% (35/40) were thrombocytopenic (PLT < 50,000/mm^3^), 47.5% (19/40) leukopenic (WBC < 4,500/mm^3^), and 77.5% (31/40) lymphopenic (< 1,500/mm^3^). In this sample population, follow-up visits were attended by 30 participants on day 50 (D50) and by 12 individuals on day 180 (D180). The reduced number of returning participants was mainly due to the difficulty of locomotion within the Amazon forest region and, in some cases, because individuals had moved to other cities. Among those who returned, 13.3% (4/30) on D50 and 50% (6/12) on D180 tested positive for malaria. Of these individuals, 60% were male and 40% female, with ages ranging from 20 to 68 years (mean: 42.5 years). In individuals from Boa vista sample were collected in D0 and D3 after initial treatment. The complete demographic data is available in study of Salazar et al, 2024 (24).

### 3.2 Recombinant expression, purification and confirmation of the Pv_LISP-2 proteins

Two distinct regions of the PvLISP-2 protein were selected based on previous reports indicating that the protein contains an N-terminal region harboring a *Plasmodium* export element (PEXEL) motif (Pv_L.seq1), which mediates protein export, as well as a central repeat region (Pv_L.seq2) that is conserved across *Plasmodium* species. Both regions exhibited high predicted antigenicity, with VaxiJen scores of 1.26 for PvLISP2_Seq1 and 2.18 for PvLISP2_Seq2. Both recombinant proteins were successfully expressed in *Escherichia coli* Shuffle T7 cells. And the predicted molecular masses of expressed proteins were 54.35 kDa and 40.46 kDa, respectively. However, the predicted molecular weights considering the percentage of acidic amino acids were 66.36 and 52.42 kDa (25) which were confirmed according to the migration pattern in the SDS-PAGE (**Figure 1B**). Both proteins were expressed in the soluble form. The purity of the recombinant protein was assessed by Western blot assay (**Figure 1C**). The results demonstrated that the expressed proteins were efficiently purified; however, an additional band was observed for Pv_L.seq2, likely corresponding to degradation products, truncated forms, or incomplete bacterial expression. The purified protein was further tested for immunoreactivity using a pooled serum from malaria-infected individuals. Western blot analysis confirmed the recognition of the specific protein bands by the patient’s sera (**Figure 1D**).

### 3.3 Anti-Pv_LISP-2 IgG and IgM antibodies are induced during malaria infection

**Figure 2:**
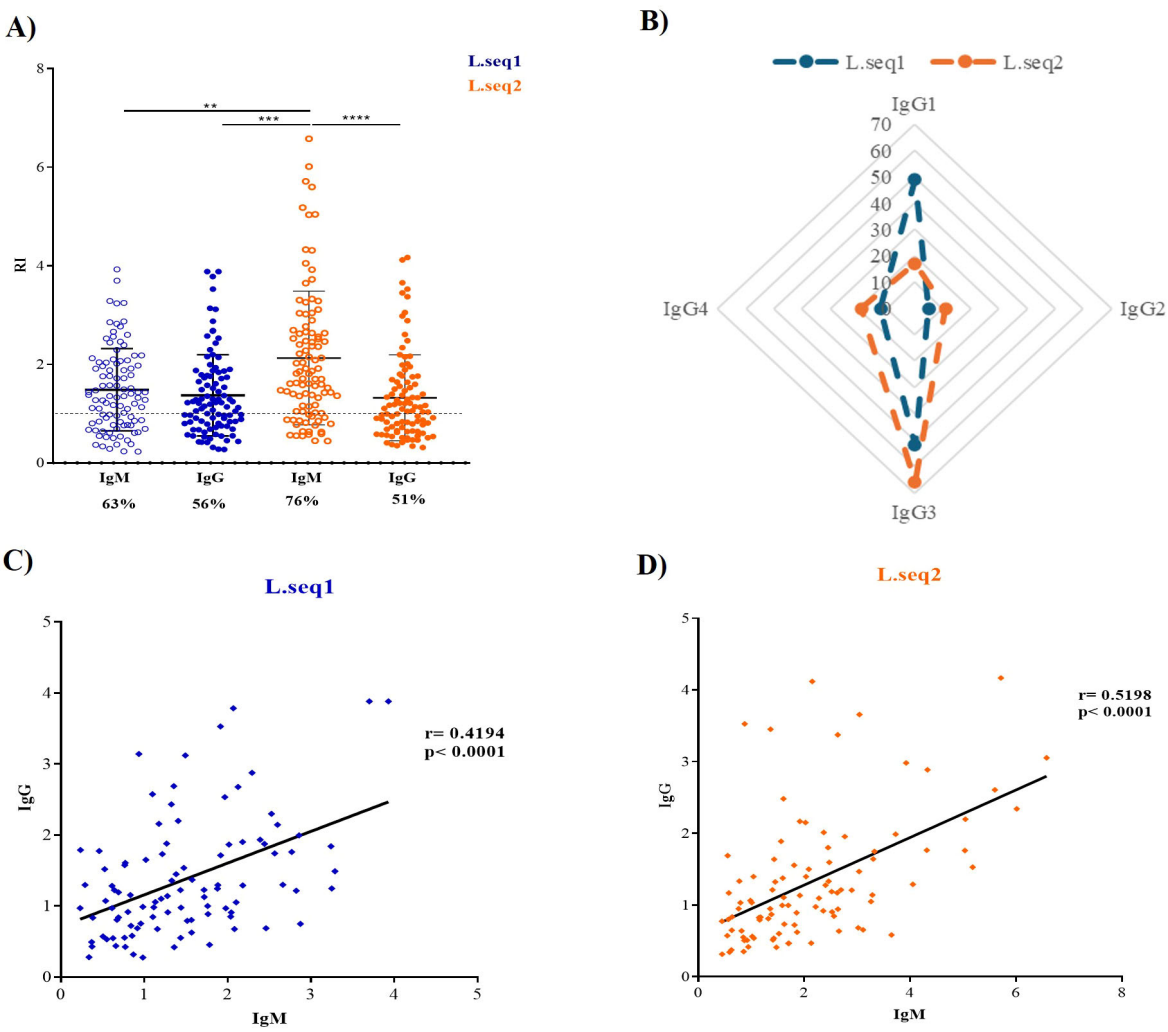
Prevalence of anti-Pv_LISP-2 antibodies and association between IgG and IgM response in individuals from Boa Vista. **(A, B)** - Reactivity index (RI) for IgM and IgG between the two different sequences of the Pv_LISP-2 antigen. The dashed line represents the cut-off that separates responders from non-responders and circles indicate the response of each individual. **(B)** Radar charts represent the percentage of individuals responsive to the IgG1, IgG2, IgG3, and IgG4 antibodies for L.seq1 (blue) and L.seq2 (orange). **(C, D)** Correlation between the reactivity index (RI) of the anti-IgG and IgM response between the two different antigens. Differences between groups were assessed using the Kruskal–Wallis test followed by Dunn’s multiple-comparison test. Associations between variables were evaluated using Spearman’s rank correlation. Data from independent experiments are presented as mean ± standard deviation. Differences were considered significant when p<0.05. Asterisks represent **p<0.01 and ****p < 0.0001.

The recombinant proteins were used to determine the presence of naturally acquired antibodies in malaria-infected individuals. In plasma samples of patients from Boa Vista (BV) during the acute phase, a higher titer of IgM than IgG was found, but only statistically significant to Pv_L.seq2 (p<0.0001) (**Figure 2A**). A moderate positive correlation between IgM/IgG was found between the responses for Pv_L.seq1 (r=0.4194 p<0.0001) and Pv_L.seq2 (r=0.5198 p<0.0001) (**Figures 2C, D**).

Next, we evaluated the predominant IgG subclasses (IgG1, IgG2, IgG3, and IgG4) in patients who were IgG-positive during the acute phase. In BV individuals, responses to L.seq1 were detected as IgG1 in 49%, IgG2 in 5%, IgG3 in 52%, and IgG4 in 12% of patients, whereas responses to L.seq2 were observed in 17%, 11%, 66%, and 19%, respectively (**Figure 2B**).

**Figure 3:**
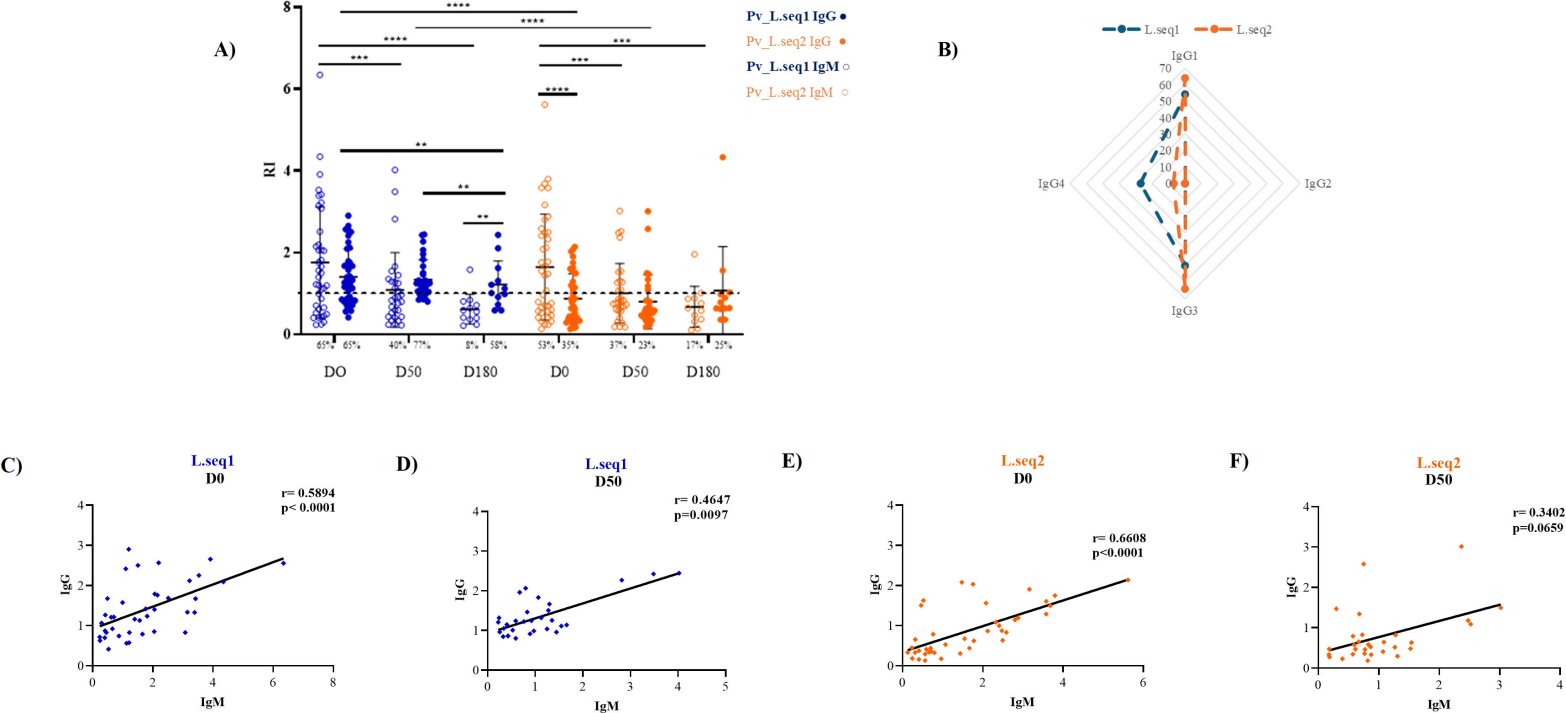
Profile of the IgG and IgM antibody response against Pv_LISP-2 during the acute (D0) and convalescent phase (50- and 180-days post-infection) of *P. vivax* infection in individuals from Manaus. **(A)** Reactivity index (RI) for IgM and IgG between the two different sequences of the Pv_LISP-2 antigen. The dashed line represents the cut-off that separates responders from non-responders and circles indicate the response of each individual. **(B)** Radar plot represents the percent of individuals responsive to the IgG1, IgG2, IgG3, and IgG4 antibodies for L.seq1 (blue) and L.seq2 (orange). Correlation between the reactivity index (RI) of the anti-IgG and IgM response in D0 **(C, E)** and D50 **(D, F)**. Longitudinal antibody responses were analyzed using linear mixed-effects models, with post hoc multiple comparisons performed using the Benjamini–Hochberg test. Correlations were tested by the Spearman test. Data from independent experiments are presented as mean ± standard deviation. Differences were considered significant when p<0.05. Asterisks represent *p<0.05 and **p<0.01.

For the Manaus population, higher IgM levels were observed during the acute phase (D0) for both Pv_LISP recombinant proteins (**Figure 3A**). IgM responses declined significantly at later time points, with significant differences detected between D0 and D50, as well as between D0 and D180, for both Pv_L.seq2 and Pv_L.seq1 (p < 0.001 and p < 0.0001, respectively) (**Figure 3A**). In contrast, IgG responses were maintained throughout the entire follow-up period, with no significant differences observed among D0, D50, and D180 for either recombinant protein. Although IgG levels appeared higher than IgM at later time points, this difference reached statistical significance only at D180 for Pv_L.seq1 (p < 0.01) (**Figure 3A**).

Like the BV sample population, a moderate positive correlation was found between IgM/IgG responses at D0 and D50, and not statistically significant in D180 (**Figures 3C–F**).

A similar pattern of IgG isotypes was observed in the Manaus population; the frequencies of responders to Pv_L.seq1 were 64%, 0%, 64%, and 7% for IgG1, IgG2, IgG3, and IgG4, respectively, and for Pv_L.seq2 were 54%, 0%, 50%, and 27% (**Figure 3B**). In summary, the results showed that patients responded weakly to IgG2, moderately to IgG4, and strongly to IgG1 and IgG3 subtypes.

As an exploratory analysis, we compared IgM and IgG levels during the acute phase across the different populations. For Pv_L.seq1 antigen, there is no difference between the two populations, however, for Pv_L.seq2 was found a higher reactivity was found in individuals from BV compared to Manaus region for both IgM and IgG, but only statistically significant for IgG (p<0.01) (**Supplementary Figure 1**).

### 3.4 Anti-Pv_LISP-2 antibodies differ between the different proteins

The antigenicity of each portion of the Pv_LISP-2 protein was also verified in the sample population. For the Manaus population, only the IgG response differs between the two different proteins, Pv_L.seq1 was more antigenic than Pv_L.seq2. In D0 65% of the individuals had IgG response to Pv_L.seq1 and 35% against Pv_L.seq2 (p<0.0001) and in D50 77% of the patients responded to Pv_L.seq1 and 23% against Pv_L.seq2 (**Figure 3A**). The response did not differ on D180 between proteins. Strong positive correlation was found between the antigens for IgM (r=0.8337, p<0.0001) (**Supplementary Figure 2A**) and a moderate correlation in IgG (r=0.4767, p<0.001) response (**Supplementary Figure 2B**).

In BV population, a similar pattern of IgG response was found to the different antigens, 56% for Pv_L.seq1 and 51% for Pv_L.seq2 (**Figure 2A**). Nonetheless, the IgM response was higher in Pv_L.seq2 (76%) than in Pv_L.seq1 (63%) (p<0.01) (**Figure 2A**). Similar to Manaus population, a strong positive correlation was found between the antigens for IgG (r=0.7044, p<0.001) (**Supplementary Figure 2C**) and IgM (r=0.7798, p<0.0001) (**Supplementary Figure 2D**).

### 3.5 Seroconversion of the anti-Pv_LISP-2 antibodies after a long-term follow-up

**Figure 4:**
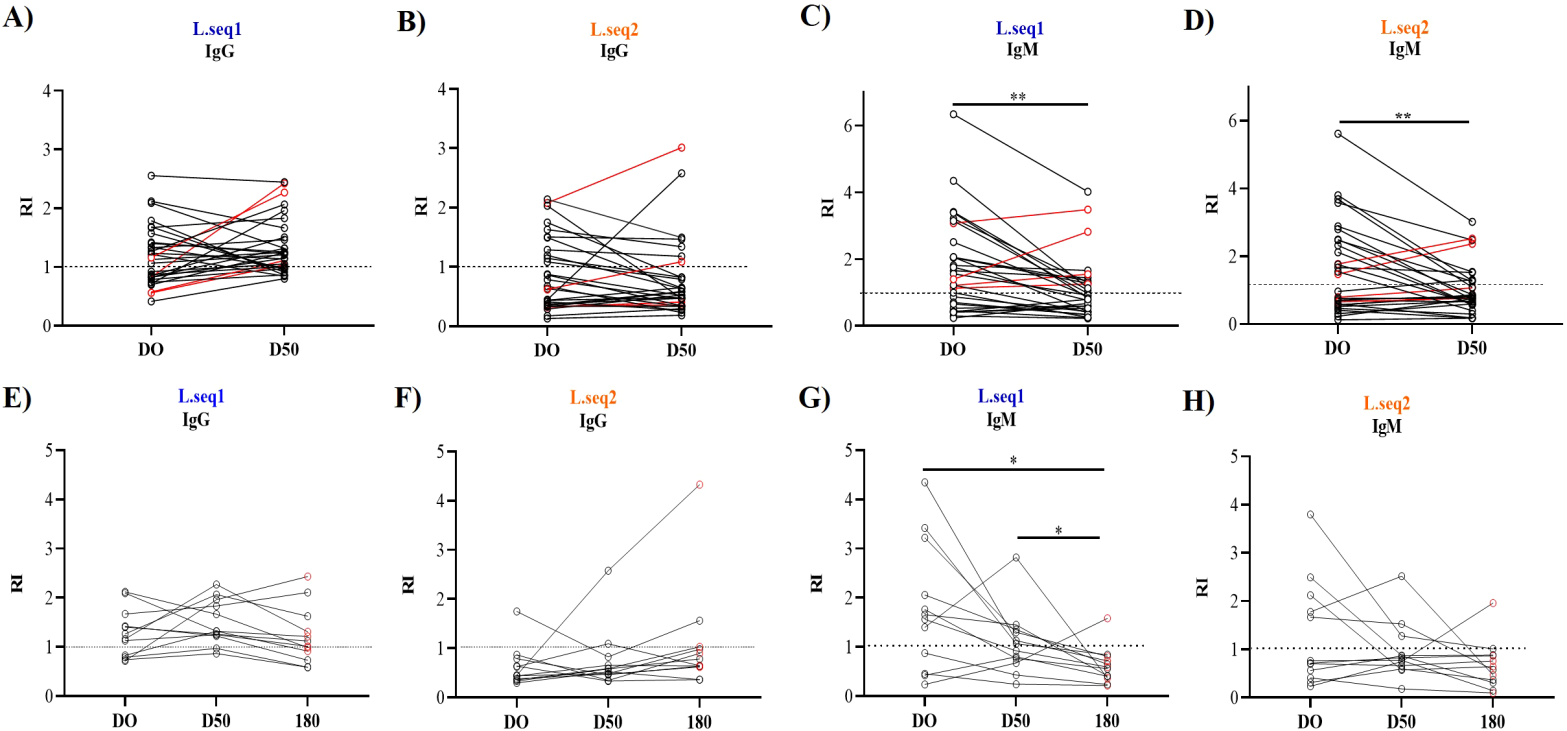
Dynamics of IgG and IgM response during acute (D0) and convalescent phase (Conv 50 dpi and 180 dpi) of vivax malaria for Pv_LISP-2. Plasma samples from patients which attended 50 days follow-up were analyzed to measure the IgG **(A, B, E, F)** and IgM **(C, D, G, H)** levels against the Pv_LISP-2. The dashed line represents the cut-off that separates responders from non-responders. Each line with spheres at the ends represents an individual (n = 30) or (n=12). Red circles represent patients who tested positive for malaria again in D50 or D180 collect. Statistical significances were tested using the Kruskal–Wallis with Dunn’s test or Wilcoxon test. Asterisks represent *p<0.05, **p<0.01.

In the Manaus cohort, we evaluated seroconversion and the presence of naturally acquired antibodies against Pv_LISP-2 over a 50-day period following infection in 30 patients. For Pv_L.seq1, 51% (n = 15) of individuals were IgG-positive at D0. Among these, 40% (n = 12) maintained their IgG response from D0 to D50, and no reinfection was observed in these patients. In addition, 33% (n = 10) seroconverted by D50, including three patients who were parasite-positive again (**Figure 4A**). In contrast, for Pv_L.seq2, only 37% (n = 11) were positive at D0, 17% (n = 5) maintained positivity at D50, and just 7% (n = 2) seroconverted (**Figure 4B**).

A distinct response pattern was observed for IgM antibodies. For the Pv_L.seq1 protein, 57% (n = 17) were IgM-positive at D0, and 36% (n = 11) remained positive at D50. All patients who were parasite-positive at D50 (n = 4) also remained IgM-positive for Pv_L.seq1 (**Figure 4C**). Similarly, for Pv_L.seq2, 45% (n = 14) were IgM-positive at D0, and 33% (n = 10) remained positive at D50, including two individuals who had tested negative at baseline (**Figure 4D**). When comparing responses between D0 and D50, only the IgM response showed a statistically significant difference (p < 0.01) for both antigens (**Figures 4C, D**).

Over 180 days, antibody kinetics could be followed in only 12 patients. During the acute phase, 67% (8/12) of individuals were IgG-positive for Pv_L.seq1. In addition, two individuals who were Pv_LISP-2–negative at D0 seroconverted by the convalescent phase, resulting in 10/12 IgG-positive individuals; among these, two were parasite-positive at D50. At D180, 70% (7/10) of those IgG-positive at D50 remained antibody-positive, and four of these seven individuals were parasite-positive at the time of sample collection (**Figure 4E**). In contrast, for Pv_L.seq2, only 8.3% (n = 1) of patients were IgG-positive during the acute phase, 17% (n = 2) seroconverted at D50, and 25% (n = 3) were positive at D180, of which 67% (2/3) were also malaria vivax positive (**Figure 4F**).

The longevity of IgM antibody assessed for both recombinant proteins showed that Pv_L.seq1 67% (n=8) were positive on DO, 50% (n=6) kept positive on D50 and only 8% (n=1) on D180, this patient was positive for malaria (**Figure 4G**). For Pv_L.seq2 42% (n=5) were positive on D0, 25% (n=4) on D50 and 17% (n=2) on D180, one of the patients was also positive for malaria (**Figure 4H**). A significant difference was observed only for IgM antibodies against Pv_L.seq1 (p < 0.05) when comparing D0/D180 and D50/D180 (**Figure 4G**).

### 3.6 Anti-Pv_LISP-2 antibodies increase with days of symptoms and IgM is correlated with parasitemia

**Figure 5:**
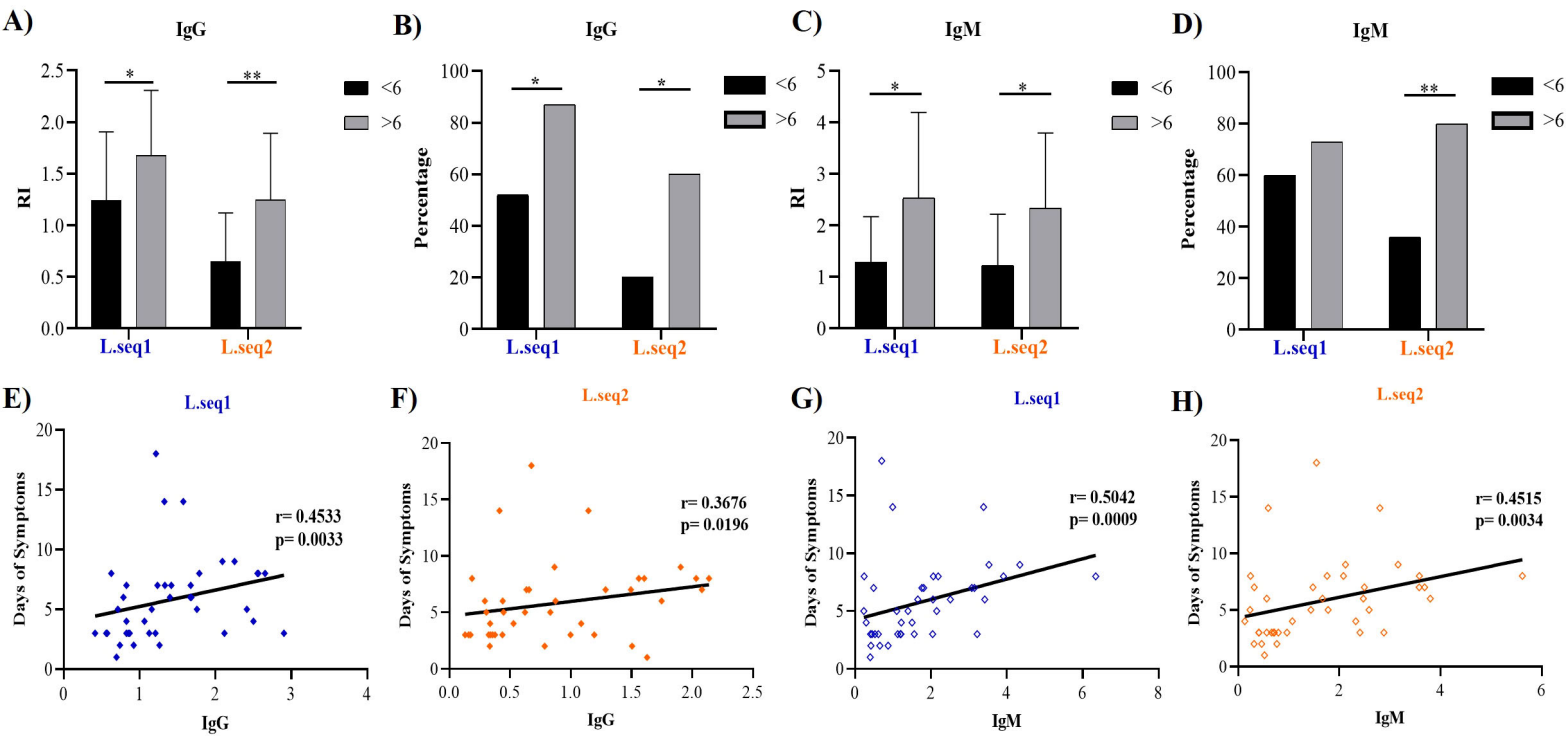
Associations between antibody responses to the recombinant antigen and days of symptoms. Patients were stratified into two groups based on days of symptoms (<6 and >6 days). IgG **(A, B)** and IgM **(C, D)** responses against the Pv_LISP-2 antigen were compared between groups. Furthermore, correlations between antibody levels and days of symptoms were evaluated for IgG **(E, F)** and IgM **(G, H)**. Differences among proportions of responders to each recombinant protein were analyzed using the Chi-square test for trend, whereas differences in the magnitude of response were evaluated using Mann-Whitney test. Correlations were tested by the Spearman correlation test. Differences were considered significant when p<0.05. Asterisks represent *p<0.05 and **p<0.01.

We evaluate the correlation of days of symptoms and the immune response against Pv_LISP-2 in patients during the acute phase. We divided patients into two different groups: less than 6 days of symptoms or more than 6 days of symptoms. The magnitude of the IgG antibody differs for Pv_L.seq1 (Mean RI: 1.2864 (Confidence Interval (95% CI), = [0.9826-1.5030]) against 1.6764 (CI95% = [1.3568-1.9960]), p<0.05) and Pv_L.seq2 (Mean RI: 0.6498 (CI95% = [0.4656-0.8340]) against 1.2453 (CI95% = [0,896-1,560], p<0.01) (**Figure 5A**). The moderate positive correlation confirms the association between the parameters Pv_L.seq1 (r=0.4533, p=0.0033) and Pv_L.seq2 (r=0.3676, p=0.0196) (**Figures 5E, F**). For both IgG and IgM responses, the percentual of positive patients increases with the days of symptoms. 52% of the patients with less than 6 days were positive to IgG in Pv_L.seq1 against 87% in patients with more than 6 days of symptoms (p<0.05). In Pv_L.seq2, 20% were positive in less than 6 days of symptoms against 60% (p<0.05) (**Figure 5B**).

Like IgG response, the RI for IgM was significantly higher in patients with more than 6 days of symptoms for Pv_L.seq1 (Mean RI: 1.2905 CI95% = [0.926-1.654] vs Mean RI: 2.4357 CI95% = [1.614-3.454], p<0.05) and Pv_L.seq2 (Mean RI: 1.2261 CI95%= [0.817-1.635] vs Mean RI: 2.3383 CI95%= [1.530-3146], p<0.05) (**Figure 5C**). For IgM antibodies, 60% of the patients were positive for Pv_L.seq1 before 6 days of symptoms against 73% (p=0.5024) after 6 days of symptoms and for Pv_L.seq2 36% against 80% (p<0.01) (**Figure 5D**). A moderate positive correlation was also found between IgM and days of symptoms, Pv_L.seq1 (r=0.5042, p=0.0009) and Pv_L.seq2 (r=0.4515, p=0.0034) (**Figures 5G, H**).

We also analyzed the difference between the magnitude of the antibody response between anemic and non-anemic, thrombocytopenic and non-thrombocytopenic, lymphopenic and non-lymphopenic and leukopenic and non-leukopenic patients but no difference was found. However, we found a moderate negative correlation between IgM response and the levels of hemoglobin, hematocrit and red blood cells. The data are summarized in (**Table 1**).

**Table 1:** Correlation between the Reactivity Index (RI) of IgM antibody in patients in the acute phase for Pv_LISP-2 with parasitemia and hematological parameters as hemoglobin, hematocrit and red blood cells.

Parameter	Pv_L.seq1	Pv_L.seq2
Parasitemia	r= 0.3824 p= 0.0178	r= 0.2857 p= 0.0821
Hemoglobin	r= -0.4059 p= 0.0114	r= -0.3494 p= 0.0315
Hematocrit	r= -0.4903 p= 0.0018	r= -0.4179 p= 0.0090
Red blood cell	r= -0.3419 p= -0.0357	r= -0.3035 p= 0. 0640

Statistical differences were calculated using Spearman’s correlation.

### 3.7 The anti-Pv_LISP-2 IgM response is negatively correlated to hematological parameters

We also analyzed the difference between the magnitude of the antibody response between anemic and non-anemic, thrombocytopenic a non-thrombocytopenic, lymphopenic and non-lymphopenic and leukopenic and non-leukopenic patients but no difference was found. However, we found a moderate negative correlation between IgM response with the levels of hemoglobin, hematocrit and red blood cells. The data are summarized in **Table 1**.

## 4 Discussion

*Plasmodium vivax* is considered the most prevalent species that causes malaria worldwide. Due to its complex biological features, advances in prevention measures, such as vaccine development, are impaired. The most studied antigens for vaccine development against *Plasmodium* spp aim to block merozoite invasion of the red blood cells, and just a few studies focus on characterizing pre-erythrocytic and liver antigen stages. Blocking sporozoite invasion, liver development, and hypnozoite recrudescence are crucial to prevent parasite survival and to hinder clinical manifestations of the disease. In addition, the liver stage is the only stage in which sterile immunity against malaria infection has been demonstrated (26). Thus, it is an important target for vaccine development.

The Pv_LISP-2 is a protein that was suggested to be involved in the immunity acquired by natural *Plasmodium* infections (18) and it was considered a liver-stage antigen candidate. However, there are currently no studies specifically characterizing the role of this antigen in naturally acquired antibody responses, which could provide important insights into its contribution to protection against both liver- and blood-stage infection. Antibody-mediated immunity can neutralize sporozoites by blocking hepatocyte invasion, inhibit merozoite entry into erythrocytes by targeting surface antigens, and opsonize parasites to promote phagocytosis by monocytes and macrophages. In addition, complement activation has been implicated in limiting parasite growth through its action on merozoites (5, 13, 27). Together, these mechanisms contribute to the gradual acquisition of clinical immunity observed in individuals living in malaria-endemic areas. Furthermore, evaluating antigen-induced immune responses in populations with distinct genetic backgrounds and from different malaria-endemic regions may help elucidate the antigenic potential of this protein (11, 28). Here, we explore the potential of the Pv_LISP-2 antigen to generate antibodies in two different populations from the Amazon region.

In malaria, IgM is considered the first line of defense and helps parasite clearance in a complementary system-dependent manner. There is some evidence that IgM acts against blood-stage parasites, and it was associated with reducing the risk of clinical disease (29–31). In this study, a high proportion of IgM antibodies was found in both antigenic portions during the acute phase and was more prominent than IgG. IgM antibody is commonly considered to be accentuated during the initial infection and appears to increase within one week post-symptomatic infection (32, 33). In agreement with what was proposed in previous studies, the IgM response quickly decays along with the infection, and it does not change even in patients who had new malaria episodes during the follow-up study (22, 33, 34). Contrary to the previous study (30) which found that IgM response is more pronounced in patients with multiple exposures, we do not observe any correlation between previous exposure and IgM levels in the studied population.

During infection, IgM is the first antibody produced, followed by IgG. Although the IgM response declines over the course of infection, it is more pronounced during the acute phase, and we also found a positive correlation between these antibodies. This induction has also been described in longitudinal studies of *P. falciparum*, where IgM against merozoite antigens is rapidly induced and co-occurs with IgG responses in both primary and naturally acquired infections, suggesting functional roles for both isotypes in malaria immunity (29, 30, 32). We also observed a slight difference in the antibody levels in the studied population. This variation can be explained by the most recent infection in individuals from Boa Vista compared to those from Manaus, the inherent characteristics of circulating parasites or by differences in transmission dynamics.

The Pv_LISP-2 was also capable of inducing a high IgG response upon *P. vivax* infection in both populations. Several *Plasmodium vivax* antigens induce IgG responses with distinct intensities and longevities, largely depending on the antigenic target (35–39). In patients from Manaus, we longitudinally followed antibody kinetics and observed that antigen-specific IgG responses remained detectable during the follow-up period. Although IgG antibodies were detected in some individuals in the absence of detectable parasitemia, their magnitude appeared to be influenced by repeated parasite exposure. Longitudinal studies of humoral immune responses have shown that antigen-specific IgG can persist for years after the initial infection, even in the absence of new infections, potentially reflecting the activation of memory B cells or long-lived plasma cells (36, 40, 41). Notably, individuals exhibiting higher IgG titers had detectable *P. vivax* parasitemia at 50 or 180 days after the primary infection, indicating that antibody responses against this antigen may be boosted by subsequent or ongoing reinfections. Importantly, due to the limited number of individuals who remained in the study throughout all follow-up sampling points, we were unable to perform robust stratified analyses to clearly distinguish antibody persistence in the absence of antigenic stimulation from responses influenced by reinfection. Therefore, these data support the presence of antigen-specific IgG at later time points rather than definitive long-term persistence independent of re-exposure. Unfortunately, we cannot follow antibody kinetics in the BV population, and since this is the first study to evaluate naturally acquired antibodies against this liver-stage antigen, we cannot compare antibody titers for this protein with those of individuals from other populations.

Since IgG subclasses mediate distinct immune effector functions, evaluating the antibody subclass profile is crucial for understanding anti-malarial immunity against specific antigens. Therefore, we also assessed the IgG subclasses induced following exposure to *P. vivax.* We showed that the cytophilic IgG1 and IgG3 were the most frequently detected subclasses among individuals infected. Studies suggest that these subclasses are the dominant IgG subclass response and are associated with protection against the disease (42–45). Together, IgG1 and IgG3 can control malaria blood stages through Antibody-Dependent Cell-Mediated Inhibition (ADCI) and by releasing cytokines or other soluble mediators (46). In addition, it can impair pathogen growth by complement activation and opsonizing phagocytosis (47–49). On the other hand, we showed a slower proportion of individuals responding to the non-cytophilic IgG2 and IgG4 isotypes. These antibodies are less prevalent in patients with malaria, and their roles during infection are controversial. IgG2 antibodies were suggested to be predominant in unprotected subjects and to be associated with a higher risk of severe malaria infection (50–52). However, another study showed a protective role of IgG2, which may activate effector cells through FcγRIIA (53). Regarding IgG4, this antibody response has been associated with cerebral malaria and is related to a high risk of infection (51, 53, 54). In fact, the contribution of each antibody isotype to malaria infection and protective immunity has yet to be fully elucidated, and the functional relevance of IgG subclasses remains controversial. Thus, this study focused on serological profiles and did not include functional assays, precluding conclusions regarding antibody-mediated protection.

In this study, we used two different regions of the Pv_LISP-2 antigen, and the levels of antibody response vary between these different portions. The Pv_L.seq1 showed to be more antigenic and more pronounced IgG titers than Pv_L.seq2 for the Manaus population. Furthermore, antibodies against this region remained more detectable throughout the follow-up period. This indicates that two regions of the protein can stimulate the immune response in different ways, and it can be explained by the intrinsic immunogenicity of each epitope. Orito et al, 2013 (18) showed that Pv_LISP-2 is processed and exported to host hepatocytes. This fact can explain why the N-terminal region is antigenic once it is in contact with the host and can then stimulate the immune cells. On the other hand, the same study showed polymorphisms in the repeat region, suggesting that the region is under strong selective pressure by the host immune system and can help to explain the variation of the response between the different portions and geographic regions. Even though the regions showed different antigenicity, the response is still correlated, especially for the IgM subclass.

The Pv_LISP-2 expression discriminates between dormant hypnozoites and early developing parasites (55). Most of the patients with malaria relapses or reinfections were positive for this protein, which we could hypothesize that this antigen can be associated with hypnozoites reactivation, although we cannot know if the patients were reinfected or a result of the hypnozoites reactivation. Our next step was to investigate whether the presence of naturally acquired antibodies against this LS stage antigen was associated with morbidity. We showed that antibodies against Pv_LISP-2 were slightly higher after 6 days of symptoms and a positive correlation between days of symptoms and antibody titer, indicating that this can be a result of the circulating parasites at the beginning of the disease. Furthermore, most of the patients during the acute phase showed anaemia, thrombocytopenia, lymphopenia and leukopenia. Interestingly, we found a negative correlation between antibody titers and several haematological parameters such as hemoglobin, hematocrit and red blood cells. These features are frequently observed in *P. vivax* malaria and may lead to severe manifestations, such as anemia (1, 56–59). However, it is not clear why this phenomenon occurs and if it is related to antigen-specific antibodies.

In conclusion, this study describes the naturally acquired antibody response against Pv_LISP-2 in the Brazilian Amazon. Currently, there is no published data on the acquisition of naturally acquired immunity to *P. vivax* Pv_LISP-2. In this study, we showed that these two antigenic regions are naturally immunogenic. Together, our data indicate the potential of these antigens as a vaccine targeting hepatocyte infections and liver stage development. Preventing parasites from developing and progressing can promote significant advances in avoiding the progression, development and morbidity of clinical disease. Although many studies need to be carried out to explore the immunogenic role of the Pv_LISP-2, understanding the naturally acquired antibody responses in endemic populations is the first step and contributes to screening and future selection of potential antigens for a multistage vaccine.

## Data Availability

The original contributions presented in the study are included in the article/**Supplementary Material**. Further inquiries can be directed to the corresponding author.

## Supplementary material

Supplementary Figure S1 Comparison between antibody responses during the acute phase P. vivax infection in individuals from Manaus and Boa Vista (BV). Reactivity index (RI) for anti-IgG and anti-IgM against Pv_LISP-2 protein between the two studied populations. The dashed line represents the cut-off that separates responders from non-responders and circles indicate the response of each individual. Differences between groups were assessed using the Kruskal–Wallis test followed by Dunn’s multiple-comparison test. Data from independent experiments are presented as mean ± standard deviation. Differences were considered significant when p<0.05. Asterisks represent *p<0.05.

Supplementary Figure S1 Association between the level of antibody responses against the two versions of Pv_LISP-2 in malaria patients from Manaus and Boa Vista (BV). Graphs show correlations for IgG **(A)** and IgM **(B)** from Boa Vista patients, and IgG **(E)** and IgM **(D)** from Manaus patients against two different versions of the Pv_LISP-2 antigen. Correlations were assessed using Spearman’s rank correlation.
