# Supplementary figures and images for: Naturally acquired antibodies to Plasmodium vivax Pv_LISP-2, a potential liver stage vaccine antigen
Source: Front Immunol. 2026 Mar 27;17:1763514. doi: 10.3389/fimmu.2026.1763514 (PMC13065704; doi:10.3389/fimmu.2026.1763514)

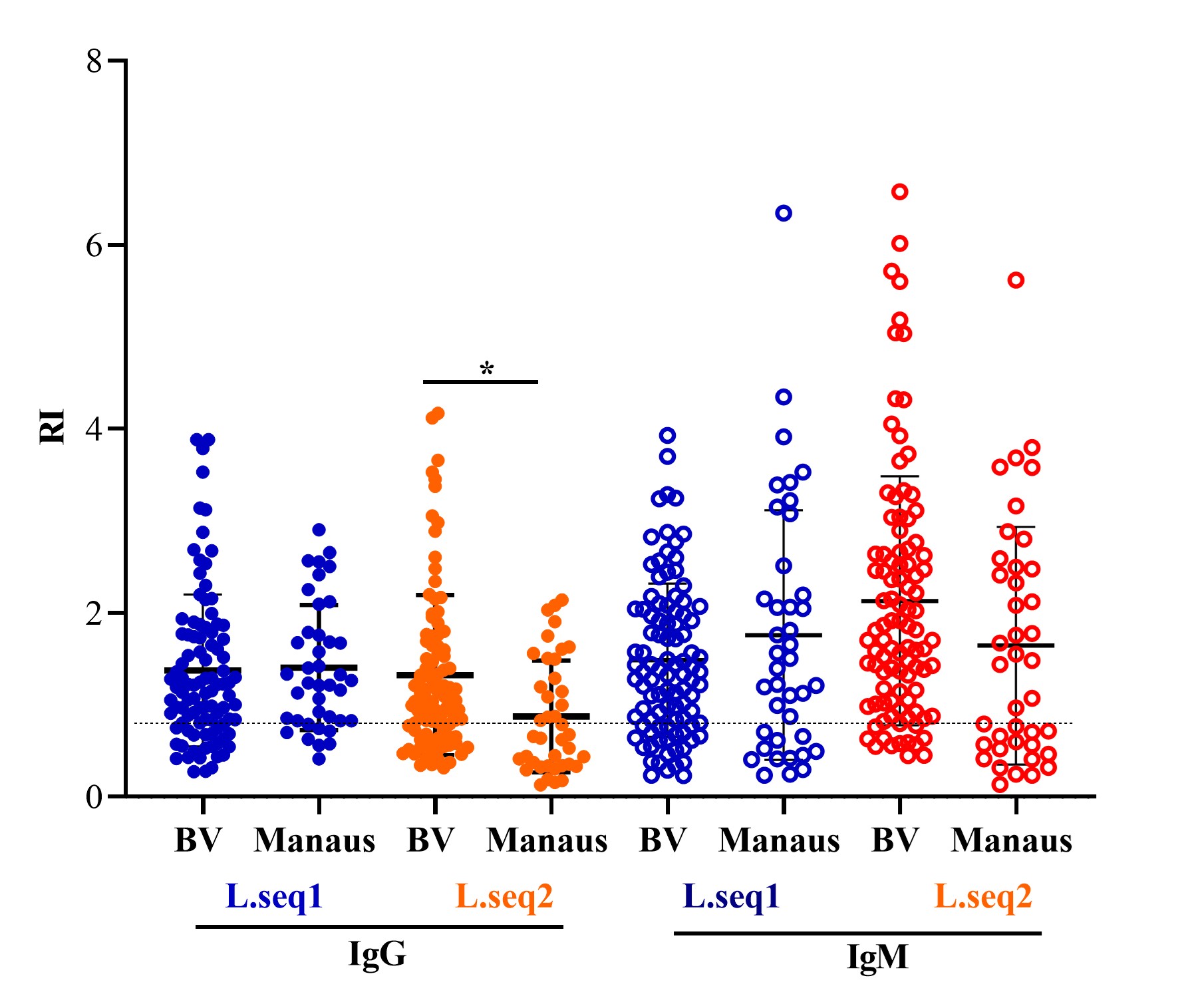

Supplement: Supplementary Figure S1 — Comparison between antibody responses during the acute phase P. vivax infection in individuals from Manaus and Boa Vista (BV). Reactivity index (RI) for anti-IgG and anti-IgM against Pv_LISP-2 protein between the two studied populations. The dashed line represents the cut-off that separates responders from non-responders and circles indicate the response of each individual. Differences between groups were assessed using the Kruskal–Wallis test followed by Dunn’s multiple-comparison test. Data from independent experiments are presented as mean ± standard deviation. Differences were considered significant when p<0.05. Asterisks represent *p<0.05. [file Image1.jpeg]

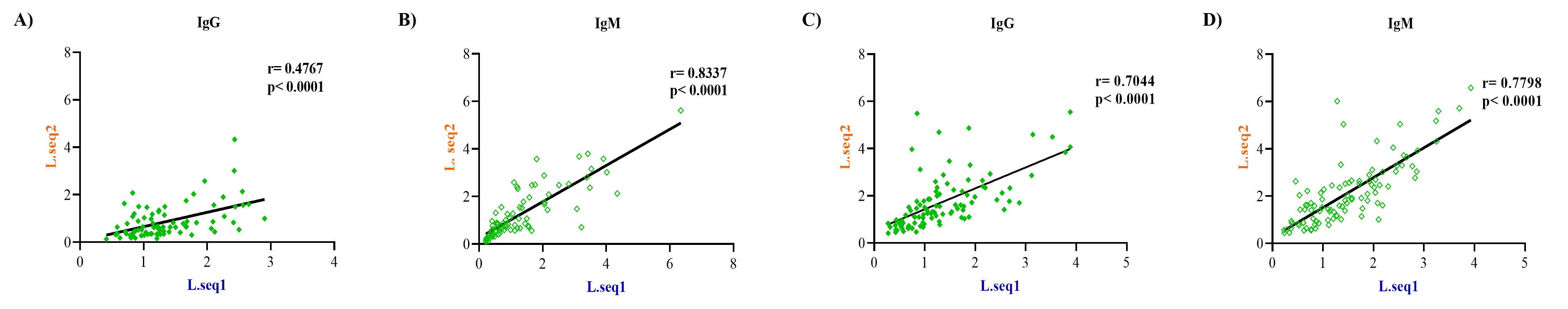

Supplement: Supplementary Figure S1 — Association between the level of antibody responses against the two versions of Pv_LISP-2 in malaria patients from Manaus and Boa Vista (BV). Graphs show correlations for IgG (A) and IgM (B) from Boa Vista patients, and IgG (E) and IgM (D) from Manaus patients against two different versions of the Pv_LISP-2 antigen. Correlations were assessed using Spearman’s rank correlation. [file Image2.jpeg]
